# The place of thoracic abdominal ultrasound influencing survival of patients in traumatic cardiac arrest imminence

**Published:** 2015

**Authors:** V Georgescu, O Tudorache, M Nicolau, G Gugonea, V Strambu

**Affiliations:** * Department of Anaesthesia and Intensive Care, “Carol Davila” Nephrology Hospital, Bucharest, Romania; ** Department of Anaesthesia and Intensive Care, “Agrippa Ionescu” Emergency Hospital, Bucharest, Romania; *** Department of Surgery, “Carol Davila” University of Medicine and Pharmacy, Bucharest, Romania

**Keywords:** thoracic abdominal ultrasound, traumatic cardiac arrest, severe trauma, algorithm

## Abstract

Severe trauma has become the most frequent cause of death in industrialized countries and, for this reason, the fastness of a diagnostic approach and the precocity of the proper treatment are both essential and best influenced by the trauma team collaboration and the existence of a specific algorithm in which each specialist has a definite place and role.

In the first stage time of a proposed specific algorithm, the vital stage, which covers the primary survey, the trauma team has not more than 5 min. (ideally) to complete airway, breathing, circulation lesions with vital potential. The ultrasound exam is placed in this stage, which is nothing more than a completion of the primary survey maneuvers, which are exclusively clinical. Two groups of patients were compared in our study; one which was named A, represented by severe traumatized patients admitted between January 2003 and December 2006 and the other one which was named B, with severe traumatized patients admitted between January 2007 and December 2012. The second group was treated by using the modified algorithm.

Although the differences were not statistically significant because of the small number of survivors, the modified algorithm was evidently superior in patients with and without cardiac arrest. If we take into account that 48 of the 261 patients survived a cardiac arrest event (although only 9 of them were discharged), the advantages of this type of algorithm are even more obvious. In lot A, 21 patients survived a cardiac arrest, of whom only 4 were discharged.

Performing an ultrasound examination during the first step of the algorithm used in the study is essential regardless of trauma causes, particularly hypovolemia. For both groups of patients with and without cardiac arrest, the percentage of patients who received ultrasound increased in the group that received a modified algorithm.

## Background 

Severe trauma has become the most frequent cause of death in industrialized countries and for this reason a fast correct approach even from the admission in the emergency department tends to be mandatory and has a maximum influence on the patient survival. The fastness of a diagnostic approach and the precocity of the proper treatment are both essential and best influenced by the trauma team collaboration and the existence of an algorithm in which each specialist has a definite place and role. Any delay in the basic maneuvers and in the clinical and paraclinical investigations that could, in turn, influence the treatment, has a fatal potential in this situation. 

This paper’s aim is to emphasize the importance of abdominal and thoracic ultrasound exam placed in the stage of the primary survey from the management algorithm for the traumatized patient.

## Material and method

The algorithm that has been used is a minimally but essentially modified one that has proved its utility in some previous and recent studies [**1**-**3**]. Mainly, the algorithm has six diagnostic and therapeutic steps which are grouped, and it is mandatory to respect this, on four time stages – I, II, III, IV. The algorithm is based on the ATLS (Advanced Trauma Life Support) algorithm elaborated by ACS (American College of Surgeons) – Committee on Trauma and has been applied in several centers proving its efficacy [**4**-**6**,**9**].

In the first time stage, the vital stage, which covers the first two steps, the trauma team has not more than 5 min. (ideally) to complete airway, breathing, circulation lesions with vital potential. The ultrasound exam is placed in this stage, which is nothing more than a completion of the primary exclusively clinical survey maneuvers. Ultrasound exam has to be very quick and efficient and, for this reason, it is not a detailed one. It can be performed in a couple of minutes and its only target is to discover the presence of hemorrhagic thoracic and abdominal sources. 

In the second time stage – 6 to 12 min. from admission – the primary survey is completed and saving life maneuvers such thoracic drainage, pericardia drainage are performed and a CT scan and/ or radiographic exams can be indicated if the patient’s status permits these actions. In fact, the first two stages of time must be completed in not more than 15 min. and cover the steps 1, 2 and 3 from the algorithm [**9**,**10**].

In the third time stage hemodynamic monitoring is completed, urinary and gastric catheters are placed, the detailed x-ray exams and blood samples analysis are performed. A reevaluation of the first steps is also performed. This lasts between 13 and 25 min. from admission.

In the fourth time stage time, which is over 25 min., special diagnostic and therapeutic procedures are performed with a stable patient. Now there are established surgical interventions except those in the primary survey, final treatment and nursing strategies.

Evaluation and reevaluation are the essential keys in this algorithm and should be performed on the entire period of management of the critical traumatized patient, as long as he remains critical.

Echocardiography is one of the most important investigations, especially in cardiac trauma. In this regard, performing it as early as possible is advisable, this type of assessment being essential for patient prognosis.

In order to highlight the importance of thoracic and abdominal ultrasound exam two groups of patients were compared, one which was named A, represented by severe traumatized patients admitted between January 2003 and December 2006 and the other one which was named B, with severe traumatized patients admitted between January 2007 and December 2012. The second group was treated by using the modified algorithm. The groups were very similar as statistical groups and the results could be compared from the statistical relevance point of view. The data were processed by using Excel 2007 and SPSS version 19 programmes. Group A consisted of 183 and group B of 387 patients.

## Results

Gender distribution of patients in the groups studied was comparative, predominantly male (74% for lot A and 76% for lot B). In the distribution of the patients in the two groups by age, it could be observed that although the absolute numbers were different, the most common average age groups were 25–34, which was predominanat in lot A and 45-54, which was predominant in lot B. They were followed by the 55-64 and 65-74 age groups, while extreme ages were less represented in the two groups. 

In the case of the classical approach of patient group A, 25 echocardiographies were performed in patients with cardiac arrest and 4 echocardiographies in patients without cardiac arrest.

**Fig. 1:**
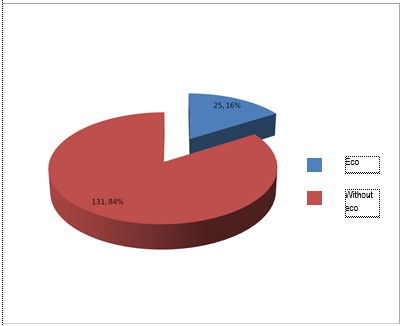
Patients with cardiac arrest and ecography - A

**Fig. 2:**
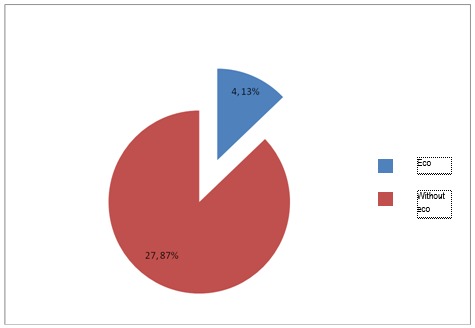
Patients without cardiac arrest and echography – A

Among patients in group A, with cardiac arrest, 16% received ultrasound examination, whilst among patients without cardiac arrest, only 13% received the same investigation.

On the group of patients with modern approach, to severe trauma, ie B group, 65 of the patients with cardiac arrest and 26 of the patient without cardiac arrest, were examined with ultrasound.

**Fig. 3:**
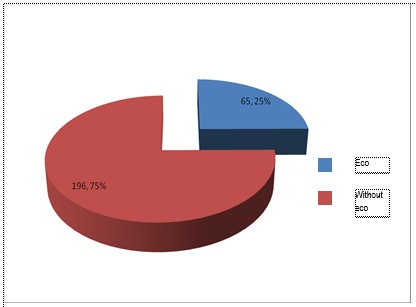
Patients with cardiac arrest and ecography – B

**Fig. 4:**
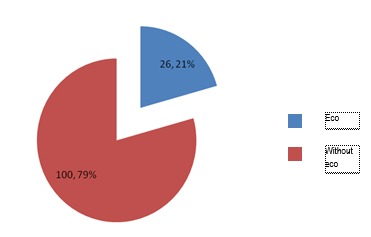
Patients without cardiac arrest and echography – B

It is noted that, taking into consideration both groups of patients, with and without cardiac arrest, the percentage of patients who received ultrasound increased for B lot, who received modified algorithm.

The chart below highlights the difference in percentage of patients examined by ultrasound between the two groups.

**Fig. 5:**
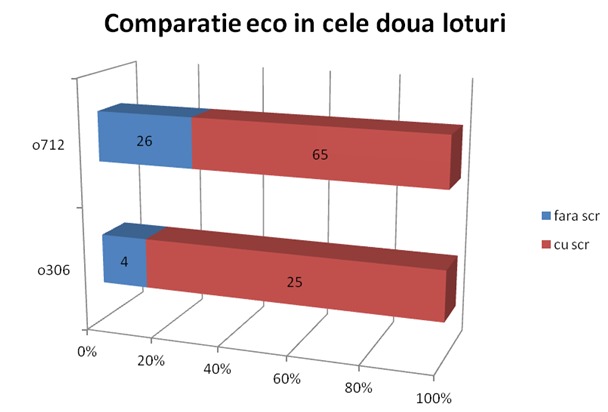
Comparative ultrasound between the two grops

In relation to the main cause of trauma in lot A, most patients who have undergone ultrasound had severe brain injury, followed by those with hypovolemia, hypoxia and hypertension pneumothorax.

As regards the duration of ultrasound, in the case lot A , it was conducted in a range of 13 to 21 minutes, and in the lot B within 7-10 minutes.The difference is statistically significant and show superiority of time staged above classical algorithm, in which ultrasound is started during the first step.

**Fig. 6:**
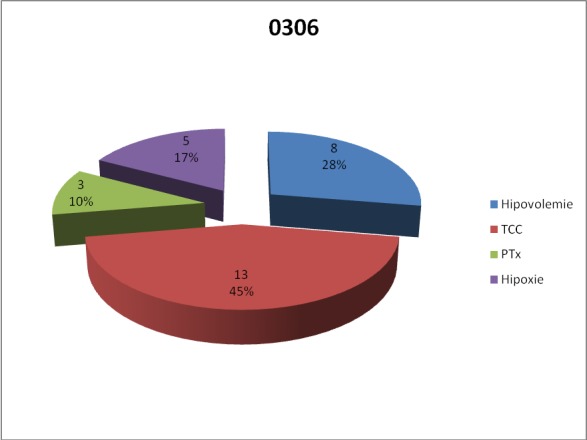
Main trauma lesions in group A

For lot B, most patients who have undergone ultrasound presented with hypovolemia as the main cause of sufference due to trauma. 

**Fig. 7:**
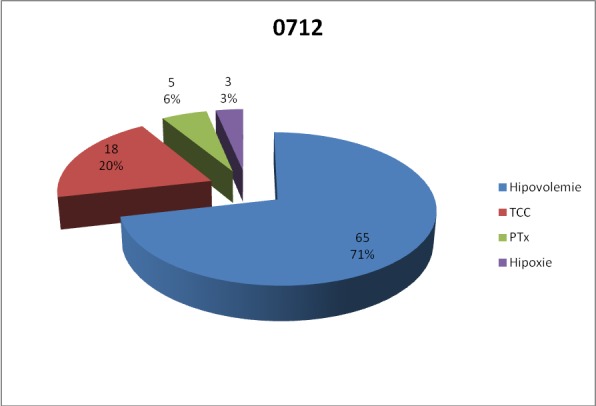
Main trauma lesions in group B

Considering that hypovolemia is present in cardiac trauma, we can say that the minuted algorithm brings a statistically significant improvement in the care of patients with cardiac trauma, which increases the chances of survival for patients suffering such trauma.

The most common causes of sufference due to trauma are, in order ,in lot A – hypovolemia, severe craniocerebral trauma, hypoxia and tension pneumothorax. In lot B, although the order of the four trauma lesions is the same, the percentages are slightly lower in the first two cases, hypovolemia and severe brain injury, and higher regarding hypoxia and tension pneumothorax.

**Figure:**
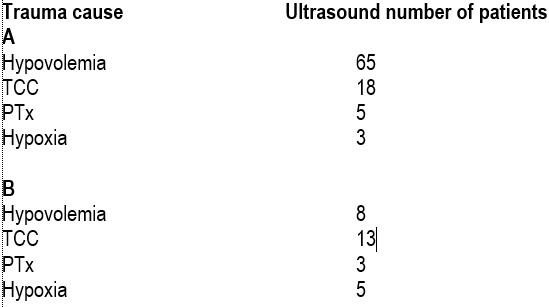


Regarding the duration of ultrasound, in the lot A it was performed in a range of 13 to 21 minutes, and in the lot B within 7-10 minutes. The difference is statistically significant and show the superiority of the minuted one compared to classical algorithm, in which ultrasound is started during the first stage.

In terms of survival, in lot A, 24 patients of those 27 without cardiac arrest have survived and only 4 patients of those 156 with cardiac arrest survived; in lot B, all 126 patients without cardiac arrest and 9 of those 261 with cardiac arrest survived. Although the differences are not statistically significant because of the small number of survivors, the modified algorithm is evident superior both in patients with and without cardiac arrest. If we take into account that 48 of the 261 patients survived a cardiac arrest event (although only 9 of them were discharged), the advantages of this type of algorithm are even more obvious. In the lot A, 21 patients survived a cardiac arrest, of which only 4 were discharged.

Among lot B survivors, 4 patients had hypovolemia caused by trauma, 2 presented with hypoxia and severe brain damage and 1 – tension pneumothorax. Among lot A survivors, 2 patients had hypovolemia caused by trauma and 2 – hypoxia.

## Discussions

Major trauma is becoming the most common cause of premature death in industrialized countries[**5**, **6**, **11**-**17**, **19**, **25**]. For this reason, a more accurate approach to patient becomes mandatory since UPU evaluation, thus having a high impact on patient survival. The speed of correct diagnosis and early starting of the proper treatment as soon as possible are both essential and dependent on collaboration between the specialists and the existence of an +exact algorithm, where each of them put their diagnostic and therapeutic experience for that time with the greatest benefit for the patient[**24**, **26**, **28**]. Late basic maneuvers ,clinical and laboratory investigations that bring additional information needed for diagnosis and choice of treatment are equally fatal.

This study tried to demonstrate just the superiority of the presence of ecography in the primary survey in both groups, but when results are compared taking into account even the time staged algorithm, the advantages are even bigger, compared with the standard approach. The first group consisted of 183 patients, and the second lot of 387 patients.

One of the most important investigations, especially in cardiac trauma is **echocardiography**. In this respect, carring it out as early as possible, this type of laboratory examinations is essential for patient prognosis. In the case of classical approach to the lot A, in patients with cardiac arrest 25 exams were performed and 4 exams in patients without cardiac arrest. In the case of the lot whre was used modern approach to the patient with severe trauma, 65 patients with cardiac arrest and 26 of those without cardiac arrest were examined with ultrasound. It can be noticed that, regarding both type of patients with and without cardiac arrest, the percentage of patients who received ultrasound increased for lot B which benefited from time staged aproach.

In relation to the main cause of trauma sufference, in group A, most patients who have undergone ultrasound had severe brain damage, followed by those with hypovolemia, hypoxia and tension pneomothorax. In the case of lot B, most patients who have undergone ultrasound had hypovolemia as the consequence of trauma, followed by severe brain damage, pneumothorax and hypoxia. Considering that hypovolemia is a marker of heart and great vessels trauma, we may say that the time staged with echography algorithm brings a statistically significant improvement in the assessment and diagnosis of patients with cardiac trauma, thus increasing their chances of survival.

In a similar study conducted in Germany, there were investigated 369 patients, 170 in group 1 classic algorithm and 199 in group 2 minuted algorithm [**5**, **6**]. Demographic data of the groups 1 and 2 were comparable in terms of age (44 +/- 20 years vs 42 +/- 23 years), male (74% vs 69%), ISS (20 +/- 18 points vs 19 +/- 15 points) and initial GCS score (11+/-4 vs 11 +/- 6 p) and no significant differences were observed [**5**, **6**].

The etiology of injuries was comparable in both groups without significant differences: the main causes of trauma were road accidents (41% vs 43%), falls from height (29% vs 30%), suicide attempts (3% vs 3 %) and violence (4% vs 3%). Other causes represented 23% in group 1 and 21% in group 2.(15,16) The type of injuries was also comparable in the two groups: cranio-cerebral injuries (59% vs 57%), extremity injuries (38% vs 37%), thoracic lesions (29% vs 30%), abdominal and pelvic injuries (19% vs 17%) and spinal injuries (16% vs 14%)[**5**, **6**].

The number of patients investigated by echocardiography (95% vs 94%) and chest X-rays (91% vs 88%) was comparable in the two groups. In contrast, the total number of patients who performed CT was higher in group 2 than in group 1 (62% vs 46%, p <0.01). After inserting the algorithm, depending on the severity of injuries, CT examinations were 85%, and emergency interventions 47% in patients with ISS> 25 [5, 6].

After inserting the algorithm, performing ultrasounds (11 +/- 10 min vs 7 +/- 6 min) and thoracic radiographs (21 +/- 12 vs 12 +/- 9 min min, p <0.01) was finished in group 2 quickly than in group 1. In addition, after entering the algorithm, ranging from patient presentation to the completion of CT examination was significantly lower in group 2 (32 +/- 14 vs 55 +/- 27 min min, p <0.01) [**5**, **6**].

In terms of survival, in our study, in patients group A, 24 of those 27 without cardiac arrest survived and also 4 of those 156 with SCR, and in the lot B, all the 126 patients without cardiac arrest survived and also 9 of those 261 with cardiac arrest. Although the differences are not statistically significant because of the small number of survivors, the second algorithm is evident superior both for patients with and without cardiac arrest. If we take into account that 48 of those 261 patients survived a cardiac arrest event (although only 9 of them were discharged), the advantages of this type of algorithm are even more obvious. In the case of lot A, 21 patients survived cardiac arrest, of which only 4 were discharged.

The present study demonstrated a correlation between the introduction of interdisciplinary treatment algorithm in emergency department, characterized by a reduction in the interval from patient presentation to the precocity of completing the diagnostic procedures and treatment initiation and staging a 4-stroke, and survival of patients with severe trauma, whether or not they suffered a cardiopulmonary arrest.

Since early therapy in resuscitation room plays a crucial role in the therapy of patients with multiple and severe injuries [**30**, **31**], introducing trauma teams has become common practice, with proven beneficial clinical effects [**20**-**23**, **4**]. In particular, studies of foreign literature have shown that the introduction of a full-time team of trauma reduced hospital mortality [**20**-**23**, **29**]. In addition, multiple trauma therapy and critical care proved to be a time management problem [**33**]. For example, 165 patients with isolated abdominal trauma proved that the probability of death increases by about 1% for every 3 minutes spent in the emergency department [**18**]. 

## Conclusions

Considering these scientific evidence, worldwide hospitals have begun to establish treatment algorithms to optimize early therapy of patients with severe trauma [**33**]. Generaly speaking, the purpose of these algorithms is to achieve fast and effective improvement of the patient's condition and set specific therapeutic and diagnostic priorities. In addition to urgent and vital performing procedures such as intubation, inserting a chest tube drainage or transfusion, is equally vital the recognition of life threatening conditions and life-threatening injuries, in order to facilitate immediate intervention without no delay [**30**, **31**, **32**]. Studies have shown that the presence of medical imaging specialist in trauma team is very important in order to interpret radiological films (especially CT) during acute condition [**32**] and performing ultrasound in the first stage of the time staged algorithm. At the same time, his presence in emergency department reduces the time spent performing ultrasonography, demonstrated by shorter duration of this type of paraclinical investigation in group of patients treated according to modern 4-stroke timed algorithm.

Performing ultrasound examination during the first step of the algorithm used in the study, is essential regardless of trauma causes, particularly hypovolemia. For both group of patients with and without cardiac arrest, the percentage of patients who received ultrasound increased for the group that received modified algorithm. In the same group, most patients who have undergone ultrasound had hypovolemia, followed by severe brain trauma, pneumothorax and hypoxia, as the cause of sufference. Considering that hypovolemia is a marker of heart and great vessels trauma, we may say that the algorithm including echography brings a statistically significant improvement in the assessment and diagnosis of patients with cardiac trauma, thus increasing their chances of survival.

Regarding duration of ultrasound, in lot A was conducted in a range of 13 to 21 minutes, and in the lot B within 7-10 minutes. The difference is statistically significant and show superiority of time staged to the classic algorithm, in which ultrasound is started during first step.

Despite the study limitations represented by the use of the algorithm in a single center of emergency and on a relatively small number of patients, the results are similar to those in the literature, and the positive effect on survival exercised by strictly timed and organized nature of the 4 stages of the algorithm, including echography is obvious. Although the data obtained should be completed and confirmed by more extensive population studies, this study could be a starting point and a model for other emergency hospitals in the country who would like to introduce such an algorithm as a standard protocol in dealing with severe trauma patient and cardiopulmonary arrest.

**Acknowledgement**

This paper is supported by the Sectorial Operational Programme Human Resources Development (SOP HRD), financed from the European Social Fund and by the Romanian Government under the contract number POSDRU/159/1.5/S/132395.
